# A Recent Suit for Malpractice

**Published:** 1873-03

**Authors:** W. F. Hutchinson

**Affiliations:** Minneapolis, Minn.


ART. II.—*A Recent Suit for Malpractice.* Reviewed by W. F. HUTCHINSON, M. D., Minneapolis, Minn.

Since it has become a settled fact that juries are to regard themselves as the avowed enemies of all medical men who may be unfortunate enough to be possessors of sufficient means to make them a mark at which unscrupulous rascals may aim their thieving attempts, it is doubtless wise to collate, from time to time, the results of the various malpractice suits throughout the country, so that the unwary doctor may recognize the danger ahead early enough to avoid the annoyance of a public trial and the loss of hard-earned dollars. One of these unhappy cases has recently been tried in Minneapolis, with the usual result of heavily mulcting the innocent physicians, who have, with slender hope of justice, asked for a new trial.

The facts are these. On the sixth or seventh of February last the plaintiff, one Getchell, at that time a laborer in the pineries, one hundred miles distant, was struck upon the right forearm by a falling tree some eight inches in diameter, suffering a transverse fracture of the ulna in the lower third and a long oblique fracture of the radius, extending from the outer surface of the bone, from a point just below the tuberosity as far downwards as the middle of the middle third, on the inner aspect. The shock was sufficient to render him partially senseless for a short time, after which he was taken to the logging camp, thence to Princeton, twenty-two miles, in a sleigh, over corduroy roads—thence to Elk River, thirty-two miles, still in sleigh, and thence to Minneapolis by rail, consuming two days on the journey. At Elk River, rough splints and a bandage were applied by a lumberman, and the limb bathed with a mixture of alcohol and saltpetre. Upon arriving hence, the arm was found badly swollen and the defendants, Drs. Hill and Lindley called upon to assume charge of the case.

The plaintiff's points were:

1st. That no attempt was made to place the bones in proper position for eight days—the arm being left upon a pillow, pronated.

2d. That due care and attention was not paid the case, visits having been made at intervals of three days, at a time when excessive inflammation necessitated close watching, and

3d. That whatever attempt had been made at repair, was at the expiration of twenty days frustrated and rendered null by the manipulation of defendants and other physicians, by them summoned in council; thereby causing non-union of the bone and leaving plaintiff a hopeless cripple.

The first witness for the prosecution, a homœopathic named T. R. Huntingdon, testified that he assumed charge of the case on March 20th; at which time there was firm union of the ulnar fracture, with partial union of the radial. On March 28th, the radial union was less than before and he firmly applied wide splints, placing the arm in the same semi-prone position in which he found it. Next saw the arm in July, after returning from an eastern trip, when the bones were entirely separated. Did not again treat the case. Saw nothing about the bone indicating wrong treatment.

The next witness, S. F. Hance, a former Buffalo practitioner, testified that he deemed it wrong, under any circumstances, to retain the arm in a prone position for twelve or thirteen days; that the interoseous space would thus be invaded, and danger incurred. Would put the arm in proper position for repair, no matter how great the swelling or inflammation. Gives as his opinion that the cause of non-union is that the bones were not placed in proper apposition, or that they were not properly treated afterward, the moving of the arm on the twelfth and thirteenth days, and on subsequent days disturbing the reparative process of nature, and that the bones will never unite now. Considers that the arm was placed in an improper position and generally ill-treated.

This witness was succeeded by several others, both regulars and homœopathic, whose testimony was in general accord with the above. The prosecution here rested.

Attorney-General Cornell opened for the defense with the evidence of Dr. Lindley, who testified that on the tenth of February, he first saw plaintiff in company with his partner, Dr. Hill. Found the arm so greatly swollen and inflamed as to be unable to discover the location of the fracture at all. Manipulation caused such extreme pain that no satisfactory examination could be made without chloroform, which was not at hand. Splints were lightly applied, and applications ordered to allay the inflammation. *The arm was placed in the easiest possible position, that being prone resting on a pillow.* This was deemed imperative, in view of the fact that the circulation in the arm was sluggish, as evidenced by the lividity and great enlargement of the limb. It was impossible to ascertain the location of the fracture before the twentieth of February, ten days after the first visit, and even then the examination was not perfectly satisfactory. The arm was placed in the semi-prone position at this time, which was as soon as could be borne.

Dr. N. B. Hill testified that the first visit was made on the tenth of February, in company with Dr. Lindley. That there was great swelling to double usual size and contusion of the limb, extending from the middle of the arm to the wrist, and that it was deemed best not to attempt reduction before the lines of fracture could be accurately defined. Obtained cloth for bandages from the ladies and made splints from thin boards—applying one to inner and one to outer surface of the arm, extending from the bend of the elbow to beyond the end of the fingers. Ordered the arm to be kept in the easiest possible position, which, after repeated trials was found to be prone, resting upon a pillow. Did not regard the reduction of the fracture as at all practicable until after the swelling had been reduced, and regarded the relief of excessive inflammatory action as the most important indication. Ordered cooling lotions and absolute rest. Visited patient again on the eleventh and thirteenth. General treatment as before, except that upon the thirteenth he put on an interoseous compress. Next visit on the sixteenth; during this time there was a gradual subsidence of the swelling; he came to the office on the twentieth, when I turned the hand up into the semi-prone position, meeting no resistance and bringing the bones nearly into position, increased tightness of bandages, retaining throughout the treatment the interoseous compress. During the fourth week, found that a provisional callus had formed, strong enough to rotate the whole shaft of the radius, and that the ulna was very firmly united; put on pasteboard splint and told him to use his fingers carefully. Six days afterwards saw arm again and found bone more firmly united. Ten days thereafter, saw plaintiff in company with Dr. Goodrich, and found that the process of repair had ceased; the union was not good. Rotation of the bone gave considerable pain, and when chloroform was suggested to make the examination painless, he refused to take it and also declined to allow any further manipulation. Plaintiff called again next day, but refused to allow of any further examination: "Saying he would not be hurt any more, was not going to have his arm broken over again, and so on." The next day after this, upon the suggestion of several physicians, a consultation at St. Paul was proposed and acceded to by plaintiff; and, after such consultation had been held, he refused to permit me to carry out the ideas of the council, which was to reapply the same dressing more tightly and wait. Told him that I wished to know if he would submit to the treatment agreed upon at St. Paul or not; that it was time to decide upon something, and that I would not be responsible as a surgeon if I could not treat the arm as I wished.

He would not submit, and left the rooms, and I did not treat him afterwards.

Several other physicians testified that they were present when plaintiff refused to allow Dr. Hill to manipulate the arm, even threatening personal violence if such were done.

Dr. Murphy, of St. Paul, testified that he saw plaintiff twice in March, and advised him to allow Dr. Hill to carry out the treatment; that there was some appearance of union, and thought that a good result might still be attained. Saw nothing wrong in any part of the treatment.

Dr. S. W. Thayer, surgeon-in-chief of the Northern Pacific Railroad, testified that he had been for more than thirty years a professor of anatomy and surgery in a Vermont Medical College; that he had heard nearly all the testimony, and had carefully examined plaintiff's arm and found an ununited fracture of the radius. If called in such a case as this is described to have been, would adopt the plan followed by Dr. Hill, viz: placing the limb in the most comfortable position until inflammation was allayed. Considered the first dressing, as well as that applied on the 20th, good surgery. Placing the hand in a prone position would not retard the process of union if not left too long. Considered that, under the circumstances, twelve days was not too long. Saw no evidence that the treatment in this case in any way retarded the union of the fractured bone.

The remainder of the testimony was concurrent with the above, every witness testifying that the surgical practice was sound, and that the process of repair had been interfered with by some cause beyond the reach of the attending surgeon.

The Attorney-General, in summing up for the defense, said that his case rested wholly upon the first twelve days' treatment, and contended that nothing had been shown proving want of care or bad surgery. That the patient had in view a suit for malpractice, incited thereto by friends (?) who did not wish him to have a good arm; that, when Dr. Huntingdon assumed charge, the callus was quite firm, with every appearance of a good result, and that nine prominent surgeons of the State agree that the treatment of the arm by Dr. Hill all the time Getchell was under his care was proper.

The attorney for the prosecution next summed up, stating his theory to be that the bones were never in apposition; that plaintiff was neglected in not being visited often enough during the first twelve days, and that the fact of so many consultations proves that the defendants knew that they were wrong. Believed that the case was established by reason of the defendants' neglect and unskilfulness.

Judge Vanderburgh then charged the jury at length. After alluding to the importance of the trial and the attention with which it had been listened to by the jury, he laid down the general principle that the plaintiff having stated his cause of action against the defendants, charging them with improper and unskilful treatment, which the defendants denied, the burden of proof was thrown on the plaintiff, the jury of course to judge as to the preponderance of evidence. He then said:

The general rule of obligation in reference to one who undertakes any office, employment, duty or trust, is that he contracts to perform it with skill, diligence and integrity.

The defendants being employed by the plaintiff as physicians and surgeons, undertook on their part that they were possessed of ordinary skill in the practice of surgery, which has been defined in the law to be such reasonable learning, skill and experience as is ordinarily possessed by others of the same profession who are in good standing as to qualifications and which reasonably qualify them to assume the responsibility of treating patients requiring surgical aid. This rule requires a proper and reasonable application. The highest skill or largest experience is not required.

Of course the defendants are required to be familiar with known rules of treatment which experience in the profession has approved as necessary in similar cases, and if an injury should result from ignorance or departure from such treatment the surgeon is responsible, unless it be made to appear also that the treatment actually adopted has also the sanction of medical men of approved skill, so that there may be reasonable ground for doubt or uncertainty as to the best mode of treatment.

2d. As to the attention, care and diligence required in the treatment employed by defendants the law requires such care and diligence as a careful and trustworthy surgeon of ordinary skill and prudence would be expected reasonably to exercise in a case of equal difficulty and importance.

3d. As to the application of the skill possessed by defendants, the law requires them to use their best skill and ability in the matter of the treatment applied, and remedies used, for the healing of the injured limb.

The foregoing in general terms embrace the terms of the defendants' obligations. Of course this responsibility rises with the importance and difficulty of any particular case. If the qualifications and treatment of this case are sufficient within the rules before stated, then the defendants are not liable, whether the result is successful or not. On the contrary, if the want of such skill or care and attention has been the procuring cause of failure to effect a cure, then the defendants are liable for such damage as nataraally and necessarily results from their malpractice in the premises.

Contributory negligence on the part of the plaintiff in order to prevent a recovery must be such as relates to the original cause of action; otherwise, if existing, it goes only to the amount of damages on the question of damaging results or aggravation of the injury.

It was the duty of the plaintiff to have co-operated with the defendants in their treatment, and to conform to their prescriptions and directions, and in case he did not, or from the presence of pain could not, he cannot hold them responsible for any consequences flowing from his, the plaintiff's, neglect or omission.

If the plaintiff, before the defendants had finished their course of treatment, refused to let them proceed further in the manner which, in accordance with their best judgment, the case required, he cannot recover, unless it is affirmatively established that the subsequent non-union of the fragments of the bone is the result of some former unskilful or negligent act of the defendants.

The jury then retired, and, after three hours' deliberation, brought in a verdict of $4,000 for plaintiff, standing eleven to one on the first ballot. Thus ended a trial wherein the great preponderance of evidence was totally ignored by a jury who had evidently prejudged the whole matter. The animus of the people at large is well shown by the following extract from the *Evening News*, published on the day following the close of the trial, and there can be no doubt that the same sentiments animated the jurors:

"At about half-past twelve this morning, the jury appeared and reported their verdict, which gave $4,000 damages to the plaintiff, Levi L. Getchell. The counsel for the defendants then moved for a new trial, and stay of proceedings was granted for sixty days.

"It is understood that on the first ballot the jury stood eleven to one for granting damages to the plaintiff. The jury then voted on the amount of damages to be given, and the vote resulted as follows: one for $500, one for $1,000, one for $3,500, two for $10,000, and seven for $5,000. Afterwards a compromise was made on the vote, and the amount of $4,000 was fixed upon as the verdict.

"The verdict seems to give general satisfaction to all but the particular class to which the defendants belong; and Mr. Getchell was noticed in the midst of a group of friends, receiving hearty congratulations. Some think that if the case is brought to trial again that the defendants will be mulcted for the whole amount claimed, instead of $4,000; but that remains to be seen. Some of the satisfaction concerning the verdict is said to come from the fact that the defendants are well able to pay the amount of the damages, or even the whole amount claimed, and that the plaintiff is a poor man. However that may be, the public are all glad that the case is over and the verdict rendered."

This ill-feeling toward the "class to which the defendants belong" is simply the same idea which led to the robberies and murders of the Paris Commune, and which is subversive of justice everywhere. For it represents simply the jealousy and hate which unsuccessful and poor men bear those who have been, through greater industry and care, more fortunate than they in amassing wealth, which feeling may at any time overrun the slender barrier of a leather-headed jury, and make itself visible in incendiary flames, or audible in shrieks for aid from burglarized citizens.

What is it but robbery to adjudge, against all evidence, the equalization of property between the doctor and his patient? when the doctor has given his best endeavors to procure a good result, and the patient, as was proven from many sources, was refractory, refusing to submit to treatment and threatening vengeance if the arm was not made as good as new. And what shall be said of those medical men who, forgetting the tie binding them to their fellows, deliberately gave such evidence as would do them greatest harm? For they must recognize the band of fraternity, and, one day, sooner or later, when it comes their turn to stand before a jury, as come it surely will, enforced remembrance of the way in which they treated brethren in the Getchell case will surely be brought home to them. The motto:

"One for all—all for one,"

must be the watchword for the profession, and just as often as it is neglected, whether for selfish motives or from fear of offense, just so often will the whole profession reap the punishment brought upon it by one cowardly and recreant member. Shoulder to shoulder the esprit du corps must be maintained, and woe to him who attacks us in the rear.

Nothing better, I think, can be found to close this article, already too long, than an extract from the charge of the Philadelphia Judge Thayer in the famous malpractice case against Prof. Reese, wherein the result, the jury being above the average of intelligence, was the discomfiture of the unscrupulous defendant:

"No presumption of the absence of proper skill and attention arises from the mere fact that the patient does not recover, or that a complete cure is not effected. God forbid that the law should apply any rule so rigorous and unjust to the relations and responsibilities arising out of this noble and humane profession! The medical man who is called to attend a patient, undertakes to possess such knowledge and skill as are usually and commonly possessed by educated physicians, and to apply that skill and knowledge with all due diligence and care for the benefit and advantage of the patient. If his performance comes up to that standard, he has discharged his duty, and is not responsible for the results. On the part of the patient, it is his duty to conform to the necessary prescriptions and treatment, if they be such as a surgeon or physicion of ordinary skill would adopt or sanction; and if he will not, or under the pressure of pain cannot, the surgeon or physician is not responsible for the injury resulting therefrom.

"When malpractice, or want of proper attention, is charged against a physician or surgeon, the burthen of proving it lies upon the person who alleges it. In the absence of satisfactory proof to establish such a charge, the presumption is that he was competent for the task which he undertook, and did his duty to the best of his ability. This is the rule of common sense, and the rule of the law upon this subject. The burthen of the proof, therefore, in this case, as in all similar cases, is upon the plaintiff. You are not to rush to conclusions detrimental to the reputation and interests of the defendant without competent proof. You are to decide the case by the evidence. You are sworn to give a true verdict according to the evidence. Your consciences must be satisfied by the evidence that the plaintiff's case is proved before you can be justified in giving a verdict against the defendant. And I will add that it is your duty to weigh the evidence carefully, and to decide the cause according to the weight of the evidence. * * * If, after looking over the whole case, and weighing all the evidence, and applying the rules of law regulating his responsibility, to which I referred in the commencement of my charge, you conscientiously come to the conclusion that the defendant was guilty of any negligence or want of ordinary skill and diligence, resulting in injury to the plaintiff, of course you will not hesitate to say so by your verdict. But if, on the contrary, you come to the conclusion that the plaintiff's complaint is altogether unfounded, then it concerns not only the interests of the parties in the present cause, and not only the interests of public justice, but also the established medical fame of this city (a fame established by many examples of men great and distinguished in this profession, who have here labored and died), that you put an end, so far as you can, to experiments, by unjustifiable lawsuits, against skilful, attentive and humane physicians."

